# The Impact of Left Ventricular Assist Device Outflow Graft Positioning on Aortic Hemodynamics: Improving Flow Dynamics to Mitigate Aortic Insufficiency

**DOI:** 10.3390/biomimetics8060465

**Published:** 2023-10-01

**Authors:** Zhuohan Gu, Chi Wei Ong, Yongzhen Mi, Ashwin Seetharaman, Ryan Ruiyang Ling, Kollengode Ramanathan, Hwa Liang Leo

**Affiliations:** 1 Department of Biomedical Engineering, National University of Singapore, Singapore 119077, Singapore; zhuohangu@u.nus.edu (Z.G.); sashwin@u.nus.edu (A.S.); 2 School of Chemistry, Chemical Engineering and Biotechnology, Nanyang Technological University, Singapore 639798, Singapore; 3 Institute of High Performance Computing (IHPC), Agency for Science, Technology and Research (A*STAR), Singapore 138632, Singapore; mi_yongzhen@ihpc.a-star.edu.sg; 4 Yong Loo Lin School of Medicine, National University of Singapore, Singapore 119228, Singaporesurrkr@nus.edu.sg (K.R.); 5 Cardiothoracic Intensive Care Unit, National University Heart Centre Singapore, National Univeristy Health System, Singapore 119228, Singapore

**Keywords:** LVAD, computational fluid dynamics, aortic insufficiency, aortic valve

## Abstract

Heart failure is a global health concern with significant implications for healthcare systems. Left ventricular assist devices (LVADs) provide mechanical support for patients with severe heart failure. However, the placement of the LVAD outflow graft within the aorta has substantial implications for hemodynamics and can lead to aortic insufficiency during long-term support. This study employs computational fluid dynamics (CFD) simulations to investigate the impact of different LVAD outflow graft locations on aortic hemodynamics. The introduction of valve morphology within the aorta geometry allows for a more detailed analysis of hemodynamics at the aortic root. The results demonstrate that the formation of vortex rings and subsequent vortices during the high-velocity jet flow from the graft interacted with the aortic wall. Time-averaged wall shear stress (TAWSS) and oscillatory shear index (OSI) indicate that modification of the outflow graft location changes mechanical states within the aortic wall and aortic valve. Among the studied geometric factors, both the height and inclination angle of the LVAD outflow graft are important in controlling retrograde flow to the aortic root, while the azimuthal angle primarily determines the rotational direction of blood flow in the aortic arch. Thus, precise positioning of the LVAD outflow graft emerges as a critical factor in optimizing patient outcomes by improving the hemodynamic environment.

## 1. Introduction

**Figure 1:**
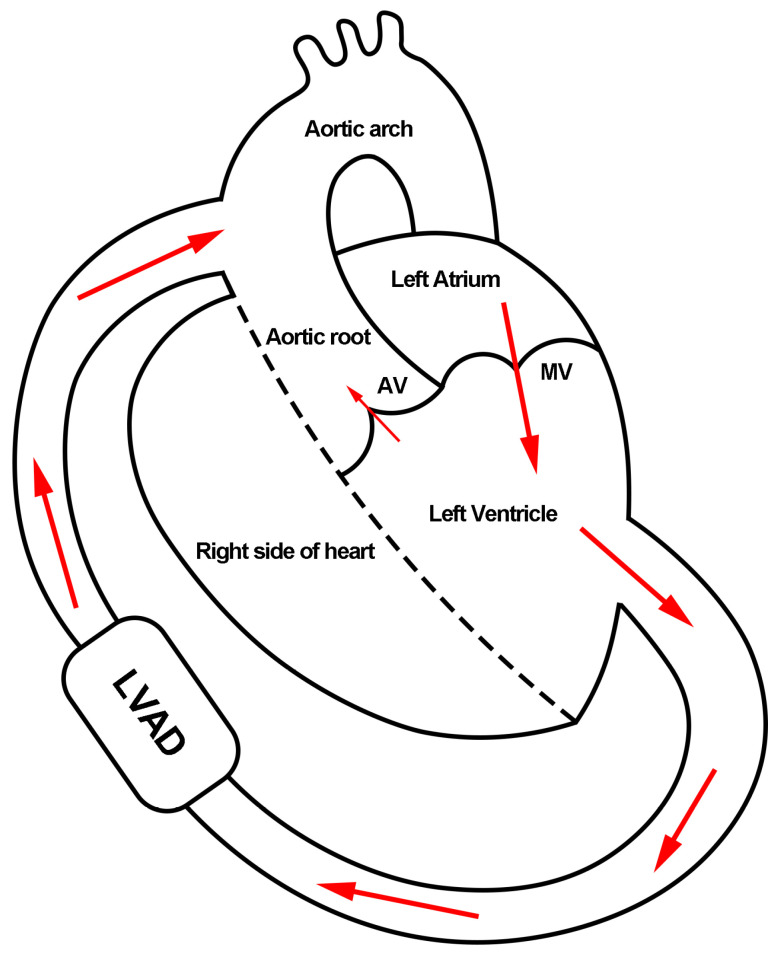
Schematic view of a heart under LVAD support. The red arrows indicate the blood flow direction in the heart. AV: aortic valve. MV: mitral valve.

Heart failure is a pressing global health issue with a steadily increasing prevalence that presently affects over 6.7 million American adults [1]. Heart transplantation, while the current gold standard therapy, is limited by the availability of donor organs [2,3]. As such, left ventricular assist devices (LVADs) have emerged as a possible alternative. Figure 1 shows a schematic view of a heart under LVAD support. With 80% of recipients surviving beyond one year, LVAD has transitioned from a temporary bridge-to-transplant support to an increasingly favored destination therapy [1,4]. More than 25,000 patients with heart failure received LVAD between 2010 and 2019, and its utilization as destination therapy increased from 34.9% to 73.1% among all patients receiving LVADs [4].

Nonetheless, LVADs are associated with potential risks, necessitating close monitoring and follow-up care [5,6]. Approximately 70% of patients experience severe device-related complications within the first year of implantation, with 80% experiencing at least one major adverse event within two years [7]. As its use as a destination therapy becomes more widespread, the complications of LVAD arising from its long-term alternations in hemodynamics, particularly aortic insufficiency, become increasingly concerning [8,9,10]. While it is known that aortic insufficiency reduces cardiovascular circulatory efficiency and causes adverse events and hospital readmissions [10], the underlying pathophysiological mechanisms remain unclear. Previous research suggests that the placement and orientation of the LVAD outflow graft anastomosis may influence flow patterns and hemodynamic parameters in the aorta, thus affecting aortic insufficiency [11,12].

Computational fluid dynamics (CFD) studies are effective engineering tools utilizing computer-based simulations to investigate fluid flow and related phenomena, providing an accessible means of investigating the hemodynamic impact of different outflow graft implantation sites [13,14]. These have increasingly focused their attention on LVAD support, aortic hemodynamics and aortic insufficiency [15,16,17]. While early studies suggest that the proximal aorta was preferred for device implantation [18,19,20], other studies have investigated how the inclination (coronal plane) and azimuthal (sagittal plane) angles [21,22,23] and cannula and jet outflow positioning affect aortic hemodynamics [24,25].

Given that a limited amount of flow occurs across the aortic valve during LVAD, previous studies have excluded the valve morphology from their simulations [10,26,27]. However, a continuously closed aortic valve may contribute to the development of aortic insufficiency [27,28,29,30,31,32], and physicians now allow for the intermittent opening of the aortic valve during LVAD support [8,33,34,35,36]. As such, the impact of the aortic valve is uncertain and should now be considered in these simulations. In this study, we incorporate the opening and closing of the aortic valve within the aortic model, and specifically analyze the hemodynamic effects of various geometric factors of the LVAD outflow cannula, including height and insertion angle. By investigating alterations in flow patterns, we aim to provide more comprehensive insights into the hemodynamic influences resulting from the insertion of the LVAD outflow cannula within the aorta.

## 2. Materials and Methods

### 2.1. Geometry Construction

**Figure 2:**
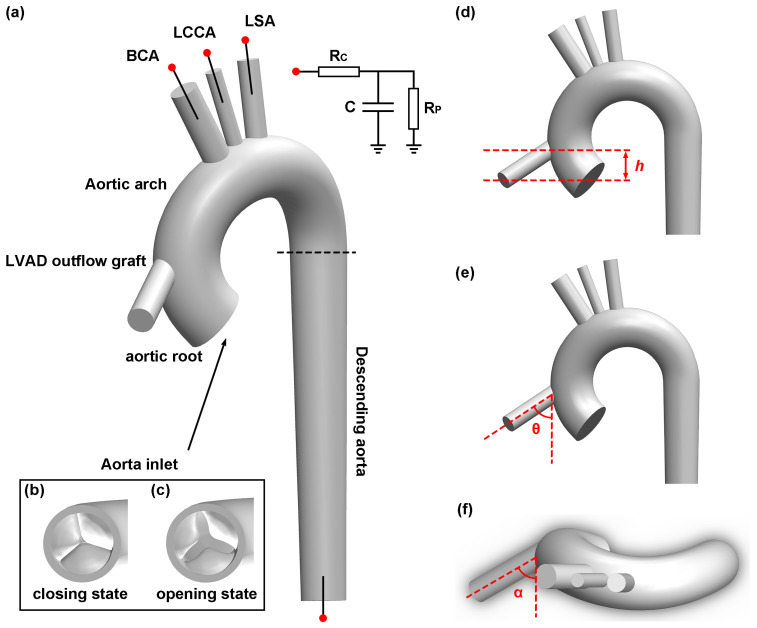
Schematic illustration of aorta geometry: (**a**) displays different part of the aorta including three epiaortic arteries, namely the brachiocephalic artery (BCA), left common carotid artery (LCCA) and left subclavian artery (LSA); (**b**,**c**) are the valve geometries in the closing and opening state; (**d**–**f**) demonstrate the definition of height *h*, inclination angle θ and azimuthal angle α, respectively, of the LVAD outflow cannula.

This study utilizes a full aorta model with curvature and angulation in the aortic arch, as previously constructed in former research based on the population-based dimension in Figure 2 [37]. The aorta model also comprises three epiaortic arteries, including the brachiocephalic artery, left common carotid artery and left subclavian artery.

The present study also employs a tri-leaflet valve model developed by three cubic T-spline surfaces in another study [38]. Two aortic valve geometries are constructed, with one model representing the aortic valve in a fully closed state with a minimal opening area and the other representing a partially opened aortic valve caused by the reduced transvalvular pressure under LVAD support in Figure 2b and 2c, respectively. This approach is adopted to show how the effective orifice area (EOA) of the aortic valve changes as blood flows through the aorta during LVAD support. 

**Figure 3:**
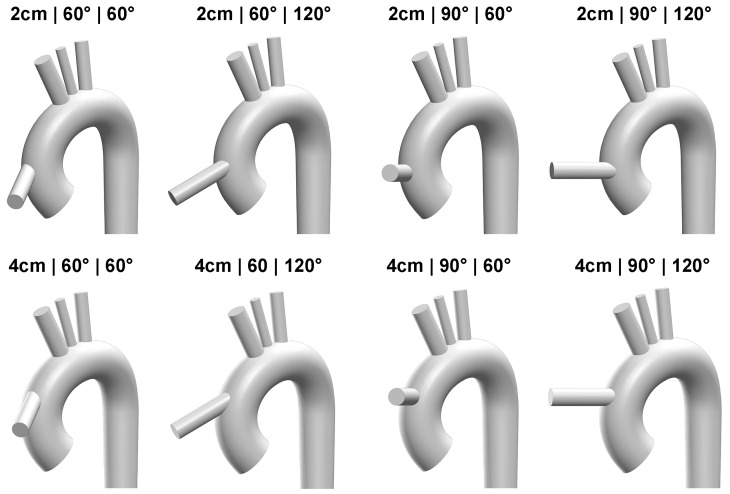
Three-dimensional view of eight different LVAD outflow graft configurations.

While some CFD studies proposed twisting the distal end of the graft to address unrealistic high-velocity flow issues, clinical evidence has indicated that kinking the graft can cause complications, including suction problems [21,39]. Therefore, the LVAD outflow graft is simplified as a straight cylindrical tube with a length of 4 cm and a diameter of 1.2 cm. The position of the LVAD outflow cannula implantation is determined by three geometric factors, namely height in Figure 2d, inclination angle in Figure 2e and azimuthal angle in Figure 2f. The azimuthal angle ranges from 60° to 120° and the inclination angle ranges from 60° to 90°. The inclination angle is set below 90° to minimize retrograde flow in the aorta. The height of the graft is between 2 cm and 4 cm above the aortic valve. In total, Table 1 presents eight cases, with two different heights, two different inclination angles, and two different azimuthal angles, as shown in Figure 3.

### 2.2. Mathematical Modelling and Governing Equations

The material property of the blood in this study is assumed to be an incompressible, homogenous Newtonian fluid with the density of 1060 kg/m^3^ and viscosity of 0.0035 Pa·s. The CFD flow simulation is based on the mass and momentum conservations for incompressible fluid given in the following equations, also known as the flow continuity and momentum equation:

(1) $$\nabla \cdot \vec{V}=0$$

(2) $$\frac{\partial }{\partial t}\left(\rho \vec{V}\right)+\rho \left(\vec{V}\cdot \nabla \right)\vec{V}=-\nabla p+\rho \vec{g}+\mu \nabla^{2}\vec{V}$$

where $\vec{V}$ is the velocity vector described by u, v, w components in x, y, z directions, p, ρ and μ stands for the pressure, density and viscosity of the fluid.

As the maximum Reynolds number of 7268 is obtained based on the peak velocity at the LVAD outflow graft inlet with the largest aorta diameter, the k–omega shear stress transport (k–ω SST) turbulence model is utilized due to its ability to accurately capture both boundary layer and free stream turbulence. This model is ideal for predicting laminar-to-turbulent transition, flow separation, reattachment, and the formation of vortices and other flow structures [40]. The pressure-based solver using the Semi-Implicit Method for the Pressure-Linked Equations–Consistent (SIMPLE–C) algorithm with second upwind orders is employed to solve all simulations. The SIMPLE–C algorithm was used for pressure–velocity coupling. The turbulent intensity is 5% and the turbulent viscosity ratio is 10 in the simulation. The convergence precision was set to 1 × 10^−5^. Four cardiac cycles with a time step of 0.0005 s are calculated in the simulation process to eliminate the numerical oscillating effect caused by the initialization in the CFD converging process and ensure a stable solution. The results of the last cycle were extracted for analysis.

### 2.3. Mesh Generation and Independence Test

The meshing process was completed by using ANSYS Fluent 19.0 (ANSYS, Inc., Canonsburg, PA, USA). Inflation layers were introduced to create thin layers near the wall and capture the rapid changes in normal gradients. The element order was quadratic, and the skewness of each model was less than 0.9 in all simulation cases. To ensure that the element size was fine enough, a mesh independence test was conducted, targeting the velocities of the three branches. The grid spacing represented by element size h was progressively decreased using a constant refinement ratio of r = 1.5. Three different meshes, namely M1 (fine), M2 (medium), and M3 (coarse), wre generated to assess the mesh quality. The averaged velocity at three outlets was extracted and the Richardson extrapolation was applied to check the mesh independence [41,42]. Based on the theory, the discrete solution f can be written in the series represented by the grid spacing h, which is:

(3) $$f=f_{exact}+\sum_{i=1}^{\infty }g_{i}h^{i}$$

where $g_{i}$ are functions defined in continuum and independent with discretization. With this assumption, the order of convergence is determined by:

(4) $$p=\ln\left(\frac{f_{3}-f_{2}}{f_{2}-f_{1}}\right)/\ln\left(r\right)$$

where $f_{1}$, $f_{2}$ and $f_{3}$ are the solutions of M1, M2 and M3. After the order of convergence is derived, the $f_{exact}$ can be calculated by:

(5) $$f_{exact}=f_{1}+\left(\frac{f_{1}-f_{2}}{r^{p}-1}\right)$$

**Figure 4:**
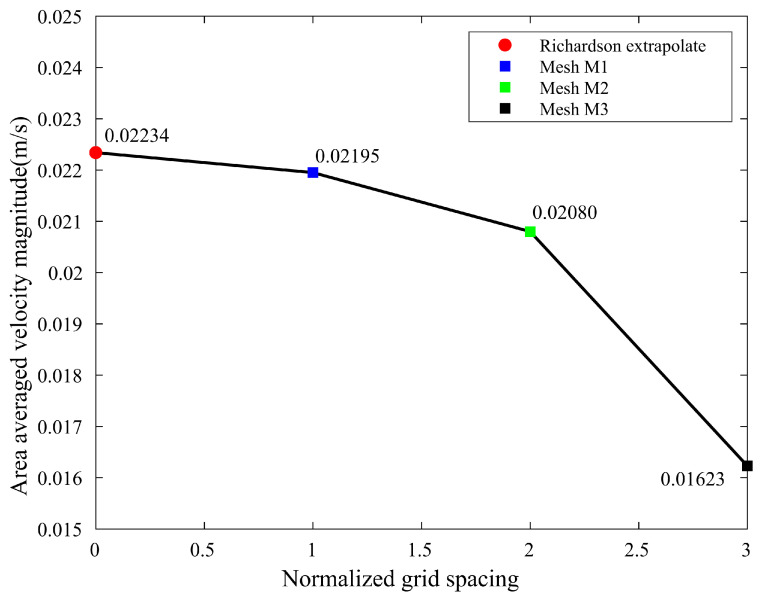
Mesh independence test conducted for three different meshes: M1, M2 and M3.

To verify the quality of the mesh, the relative error and grid convergence index (GCI) are calculated using the following formula:

(6) $$E_{1}^{fine}=\frac{f_{2}-f_{1}}{f_{1}}$$

(7) $${GCI}_{fine}=\left(\frac{F_{S}\times E_{1}^{fine}}{1-r^{p}}\right)$$

in which the safety factor $F_{S}$ = 1.25 in the simulation. By using the Richardson extrapolation method, the refinement of mesh from M2 to M1 yielded a GCI of 2.2%, which ensures the mesh independence in Figure 4. Therefore, the results obtained using the M2 mesh are mesh-independent.

### 2.4. Boundary Condition

**Figure 5:**
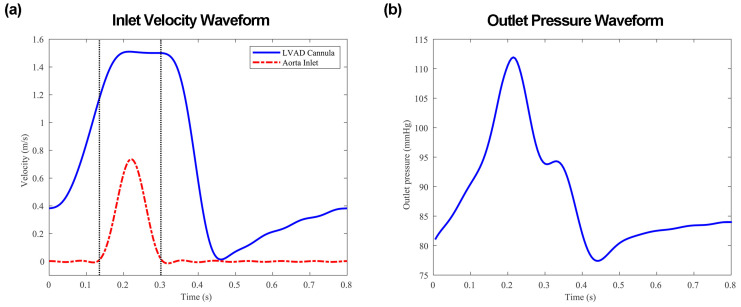
Inlet and outlet boundary conditions: (**a**) displays the inlet velocity waveforms; (**b**) reports the pressure waveform at the three epiaortic arteries.

The fluid domain comprises two inlets. The main inlet is from the LVAD outflow graft and the other one is from the aortic valve. The physiological velocity waveforms of the two inlets are validated by the clinical data in Figure 5a [43]. The rotational speed of LVAD is set to 8000 rpm and the inflow rate of the graft and aorta in the study is set to 4.5 L/min and 0.8 L/min respectively, resulting in a total cardiac output of 5.3 L/min. The inlet velocity waveforms at the aortic inlet are adjusted based on the area of the inlet surface. The cardiac cycle is set at 0.8 s with a systolic period duration of 0.3 s. Each outlet is applied with a lumped parameter model to better simulate the pressure and flow waveforms in the downstream vasculature. The three-element Windkessel model with two resistances and one compliance is chosen due to its widespread use and acceptance in accurately simulating the systemic circulation defined by the following equation [44,45]:

(8) $$\left(1+\frac{R_{C}}{R_{P}}\right)Q+CR_{C}\frac{dQ}{dt}=\frac{P}{R_{P}}+C\frac{dP}{dt}$$

where $R_{C}$, $R_{P}$ and C are the characteristic resistance, peripheral resistance, and the compliance, respectively. The parameters utilized in the model are derived from the same study that was used to construct the aorta geometry in Table 2 [37]. This boundary condition was provided by employing a user-defined function in Fluent. Figure 5b shows the pressure waveform.

The cardiac flow cycle is divided into three stages based on whether there is a flow passing through the aortic inlet. During the first stage (0 < t ≤ 0.135 s), the velocity at the aortic valve is zero, and the aortic valve is closing. During the second stage (0.135 s < t ≤ 0.3 s), the velocity at the aortic valve is greater than zero, and the aortic valve partially opens. During the third stage (0.3 s < t ≤ 0.8 s), the velocity at the aortic valve is once again zero, and the aortic valve is closing again. The results are presented at five essential time points to elucidate the transient dynamics of blood flow:

T1 = 0.1 s is the mid-acceleration of the outflow graft velocity.

T2 = 0.215 s is when the flow through the aorta reaches maximum (systole).

T3 = 0.25 s is when the flow through the aorta reaches zero.

T4 = 0.385 s is the mid-deceleration of the outflow graft velocity.

T5 = 0.46 s is when the flow through the outflow graft reaches minimum (diastole).

### 2.5. Quantities of Interest

This research mainly studies the time-averaged wall shear stress (TAWSS), oscillatory shear index (OSI) and vortex structure.

- TAWSS is applied as the temporal average of WSS vector intensity for one cardiac cycle to evaluate the wall shear stress on the aortic wall and aortic valve leaflet shown in the following equation [46]:

(9) $$TAWSS=\frac{1}{T}\int_{0}^{T}\left|\vec{\tau_{w}}\right|dt$$

where $\tau_{w}$ is the wall shear stress and T represents the time of one cycle.

- OSI is introduced to describe the degree of deviation of the WSS from its mean direction [46]. The definition of OSI is shown in the following equation:

(10) $$OSI=\frac{1}{2}\left(1-\frac{\left|\int_{0}^{T}\vec{\tau_{w}}dt\right|}{\int_{0}^{T}\left|\vec{\tau_{w}}\right|dt}\right)$$

where $\tau_{w}$ is the wall shear stress and T represents the time of one cycle. OSI ranges from 0 to 0.5, representing the transformation from unidirectional steady flow to perfectly oscillating back-and-forth velocities over the cardiac cycle.

- The vortex structure plays a crucial role in hemodynamics, as pathologically altered vortices may reduce the velocity of blood flow and lead to blood clots or thrombosis [47]. The Q-criterion method is applied due to its ability to provide a clear visualization of the three-dimensional vortex structure. The second invariant of the velocity gradient tensor Q is:

(11) $$Q=\frac{1}{2}\left({\left\|\Omega \right\|}^{2}-{\left\|S\right\|}^{2}\right)$$

where Ω is the vorticity tensor and S is the rate of strain. As the Q-criterion method defines vortices as areas where the magnitude of the vorticity is greater than the magnitude of the rate of strain, the vortex exists when Q is larger than zero [48].

## 3. Results

### 3.1. Effect of Outflow Graft Location on Velocity Distribution

**Figure 6:**
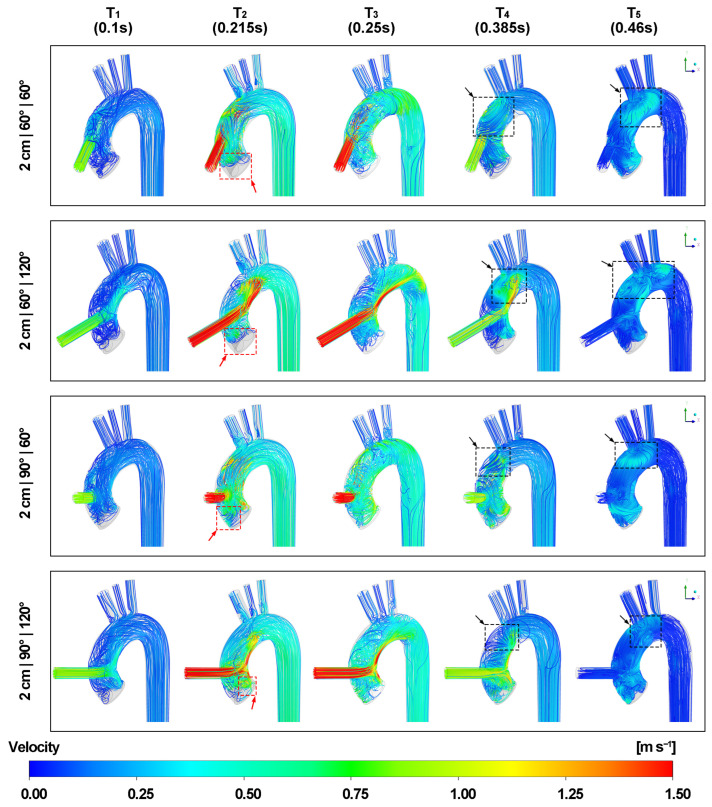
Velocity streamlines at five different time points. The black arrows with dotted boxes mark streamlines with opposite rotational directions between the graft insertion site and the brachiocephalic artery during the deceleration of the outflow graft velocity. The red arrows with dotted boxes mark the recirculation region on the aortic valve cusp.

**Figure 7:**
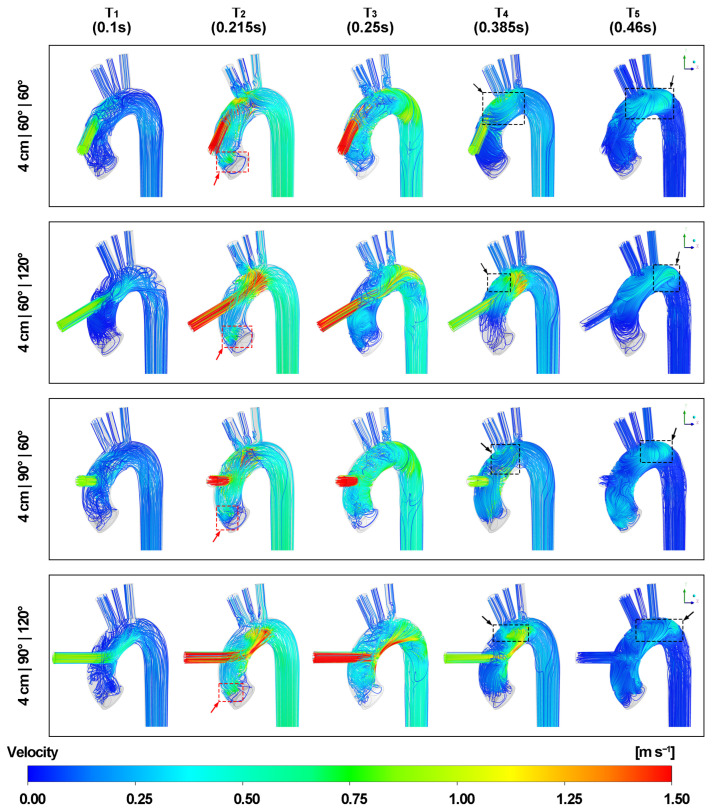
Velocity streamlines at five different time points (contd.). The black arrows with dotted boxes mark streamlines with opposite rotational directions between the graft insertion site and the brachiocephalic artery during the deceleration of the outflow graft velocity. The red arrows with dotted boxes mark the recirculation region on the aortic valve cusp.

Figure 6 and Figure 7 demonstrate the velocity streamlines of the blood flow in the three-dimensional view. The blood flow lines reveal that the jet flow from the LVAD outflow graft impinges on the inner aortic wall as the inlet velocity increases. After impingement, the graft flow splits into upward and downward bifurcated flows. Most of the blood flows directly towards the aortic arch, entering three periaortic arteries and the descending aorta. However, the remaining retrograde flow forms a recirculation region beneath the jet flow, which in turn leads to irregular flow lines at the aortic root. A secondary impingement transpires when the LVAD outflow graft is positioned close to the apex of the aortic arch. In this scenario, the blood flow impacts the distal end of the aortic arch before entering the descending aorta. A swirling flow is observed throughout the cardiac cycle at the rear end of the aortic arch as shown in the supplementary materials. The introduction of the valvular structure creates unique characteristics. Specifically, the retrograde flow at the aortic root becomes more intricate. Due to the curved structure of the aortic valve leaflets, the reverse blood streamlines follow the curvature and generate localized recirculation regions on the surface of the valve. As a result, flow lines display increased irregularities around the aortic root.

During the acceleration of velocity from the LVAD outflow graft, specifically at time points T1 and T2, a reduction in flow lines is observed at the aortic root, especially as the graft height increases. At time point T2, as the aortic inlet velocity attains its peak, the red arrows with dotted boxes show that a portion of flow lines from the inlet forms a recirculation area on the right coronary cusp, which is situated directly beneath the LVAD outflow cannula entrance. Conversely, the flow lines surrounding the other two aortic valve cusps are considerably fewer as the graft height increases. Although the maximum velocity from the aortic inlet is higher, it remains lower than the maximum graft velocity. Consequently, the velocity streamlines from the aortic inlet encircle the jet graft flow lines. Irregular flow lines are also noted at the bifurcation of the aortic arch and epiaortic arteries.

At time point T3, as the aorta inlet velocity reduces to zero and the aortic valve prepares to close, rotational flow patterns emerge at the distal end of the aortic arch, accompanied by irregularities in the flow lines within the descending aorta.

During the deceleration of graft flow velocity, specifically at time points T4 and T5, flow lines with opposite rotational directions are observed in the aortic arch between the graft insertion site and the brachiocephalic artery marked by black arrows with dotted boxes. As the graft velocity reaches its minimum at T5, these streamlines follow the curvature of aortic arch and migrate to the rear end. Furthermore, a reverse flow is observed in the LVAD outflow cannula and the flow lines in the epiaortic arteries return to the regular patterns.

### 3.2. Effect of Outflow Graft Location on Vortex Structure

**Figure 8:**
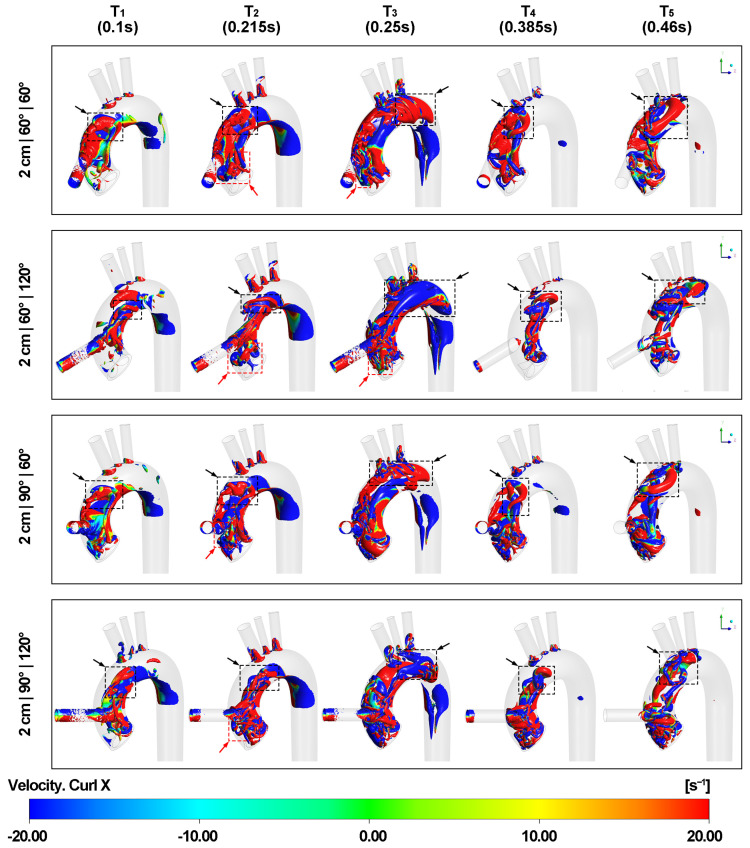
Q-criterion isosurface of vortex at five different time points. Blue means rotating in an anti-clockwise direction along the X axis according to the right-hand rule and red means rotating in a corresponding clockwise direction. The black arrows with dotted boxes show the development of the hairpin vortices into horseshoe vortices, finally affecting the rotating direction at the distal end of the aortic arch before entering the descending aorta. The red arrows with dotted boxes mark the vortex ring formed on the aortic valve due to the presence of the valve morphology.

**Figure 9:**
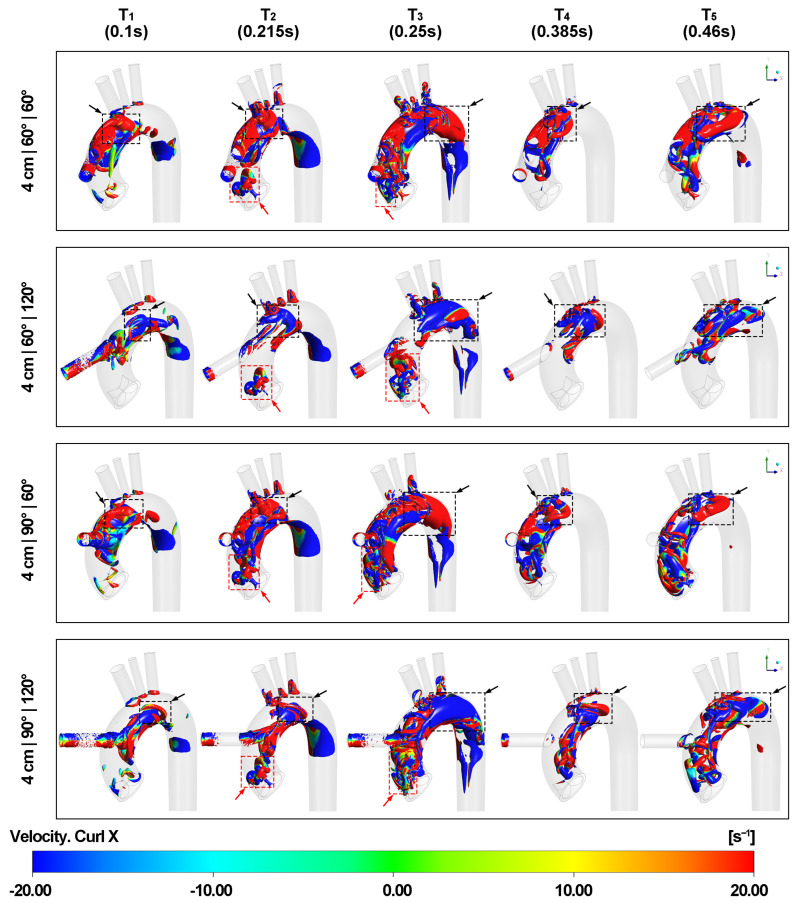
Q-criterion isosurface of vortex at five different time points (contd.). Blue means rotating in an anti-clockwise direction along the X axis according to the right-hand rule and red means rotating in a corresponding clockwise direction. The black arrows with dotted boxes show the development of the hairpin vortices into horseshoe vortices, finally affecting the rotating direction at the distal end of the aortic arch before entering the descending aorta. The red arrows with dotted boxes mark the vortex ring formed on the aortic valve due to the presence of the valve morphology.

Figure 8 and Figure 9 depict the effect of different outflow graft positions on Q level over the 0.05 isosurface of vortex formations in aorta. During the acceleration of the graft velocity at time point T1, a vortex ring forms in the aortic arch in alignment with the direction of the LVAD outflow cannula insertion. Upon impingement, the vortex expands and dissipates into hairpin vortices after colliding with the aortic arch wall. This pattern corresponds to the upward and downward bifurcated flows represented in the velocity streamline. When the LVAD outflow graft height is 2 cm, the downward hairpin vortex interacts with the edge between the non-coronary cusp and the left coronary cusp of the aortic valve due to the presence of the valvular structure. This interaction creates small eddies along the edge and curved surfaces of the aortic valve leaflets.

At time point T2, when the aortic velocity is at its maximum during the valve opening phase, another vortex ring forms at the aortic root above the aortic valve marked by red arrows with dotted boxes, corresponding to the shape of the aortic valve inlet. The contour of the secondary velocity magnitude in Supplementary Figure S2 also supports this finding. The part of the vortex ring near the inner curvature rotates in the same direction as the downward hairpin vortex, causing the two vortical structures to merge and intensify the swirling force above the edge between the non-coronary cusp and the left coronary cusp. As a result, the vortex ring becomes tilted and is no longer parallel to the ascending aorta surface. Additionally, thin vortical layers form at the inner aortic wall of the entire aortic arch and extend to the descending aorta when both graft and aortic velocities reach their maximum. At time points T2 and T3, small vortices appear at the junction of the aortic arch and the three epiaortic arteries, corresponding to irregularities in the flow lines.

Around time point T3, when the aortic valve is about to close, the tilted vortex ring from the aortic valve disintegrates, and the remaining eddy wraps around the LVAD outflow graft entrance. The asymmetrical space around the LVAD outflow cannula entrance in the aortic arch caused by different cannula locations leads to a strong vertical vortex in most cases, as shown in Figure S3. This vortex forms between the aorta inlet and the brachiocephalic artery and follows the curvature of the aortic arch. Furthermore, vortices can be observed at the distal aortic arch with the development of blood flow in the aortic arch, as shown in Figures S4 and S5.

As graft velocity decelerates at time points T4 and T5, the primary hairpin vortices transform into horseshoe vortices as multiple new hairpin vortices develop. Due to the torsion of the aortic arch, the upward horseshoe vortex expands in the top lumen of the aortic arch with spinning, which is noticeable at time point T5 when the graft velocity is at its minimum. When the LVAD outflow cannula height is 2 cm, more small eddies appear above the aortic valve because of the downward horseshoe vortex, resulting in increased turbulence at the aortic root.

### 3.3. Effect of Outflow Graft Location on TAWSS Distribution

**Figure 10:**
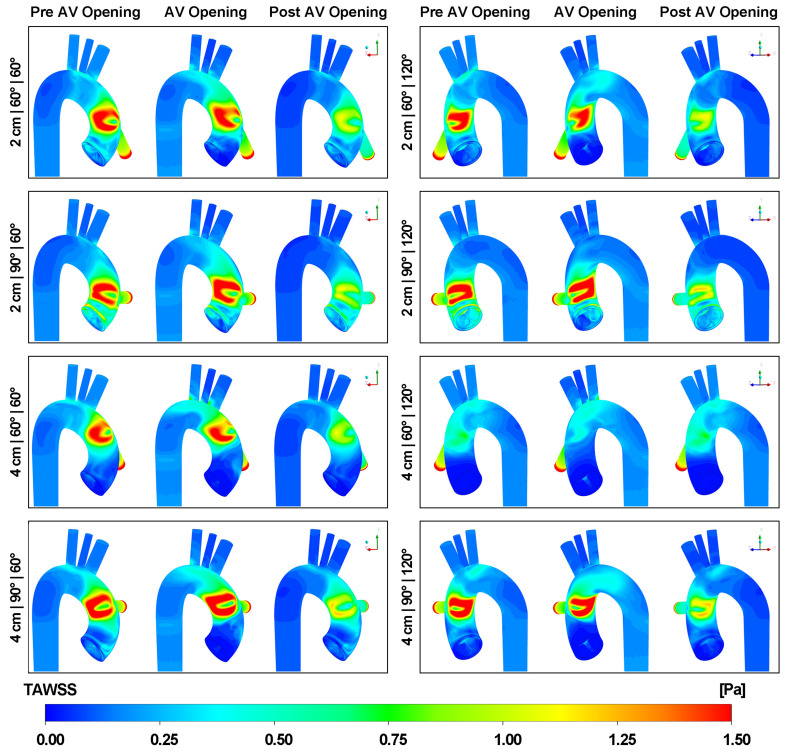
TAWSS on the aortic wall at three different stages.

Figure 10 presents the TAWSS distribution on the aortic wall. The result is presented in three critical stages, namely pre-AV opening, AV opening and post-AV opening. It is noticeable that the TAWSS on the wall of the LVAD outflow graft is higher than that on the aortic wall. This observation is because of the smaller diameter of the LVAD cannula and the comparatively high velocity of the blood flow it carries. In terms of the distribution pattern, the simulation reveals a local minimum TAWSS region sandwiched between two high-stress spots (TAWSS > 2 Pa). However, this specific TAWSS pattern is not observable in the 4 cm|60°|120° case. It is worth noting that when the aortic valve opens, the high-stress area slightly shifts upwards. Besides, the TAWSS magnitude remains low around the outlets.

**Figure 11:**
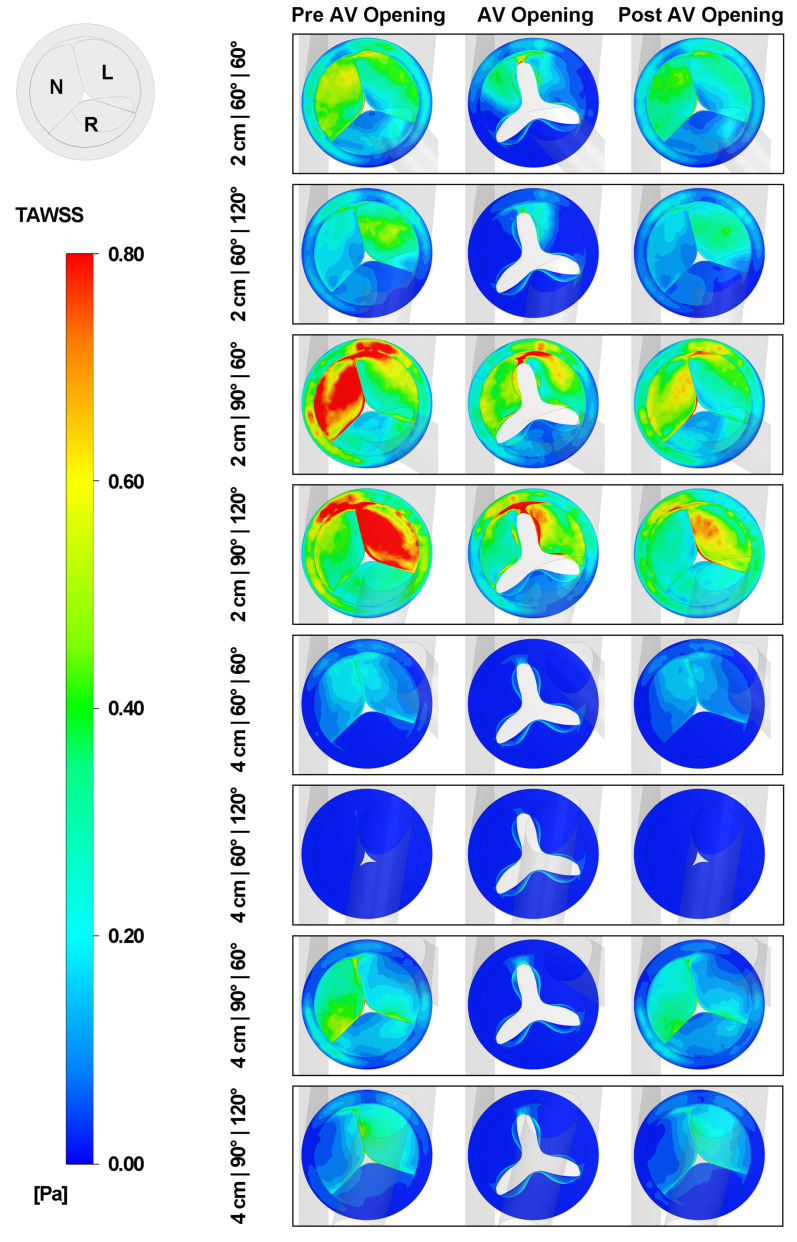
TAWSS on the aortic valve at three different stages. N: the non-coronary cusp. L: the left coronary cusp. R: the right coronary cusp.

Figure 11 depicts the TAWSS on the aortic valve at three different stages. As the height of the graft increases, the TAWSS on the aortic valve decreases for all cases. Furthermore, as the inclination angle increases, the TAWSS on the aortic valve correspondingly rises. In addition, the three leaflets of the aortic valve experience different TAWSS throughout the cardiac cycle. With an azimuthal angle of 60 degrees, the non-coronary cusp endures the highest TAWSS. However, as the azimuthal angle increases, the left coronary cusp becomes the area with the highest TAWSS.

### 3.4. Effect of Outflow Graft Location on OSI Distribution

**Figure 12:**
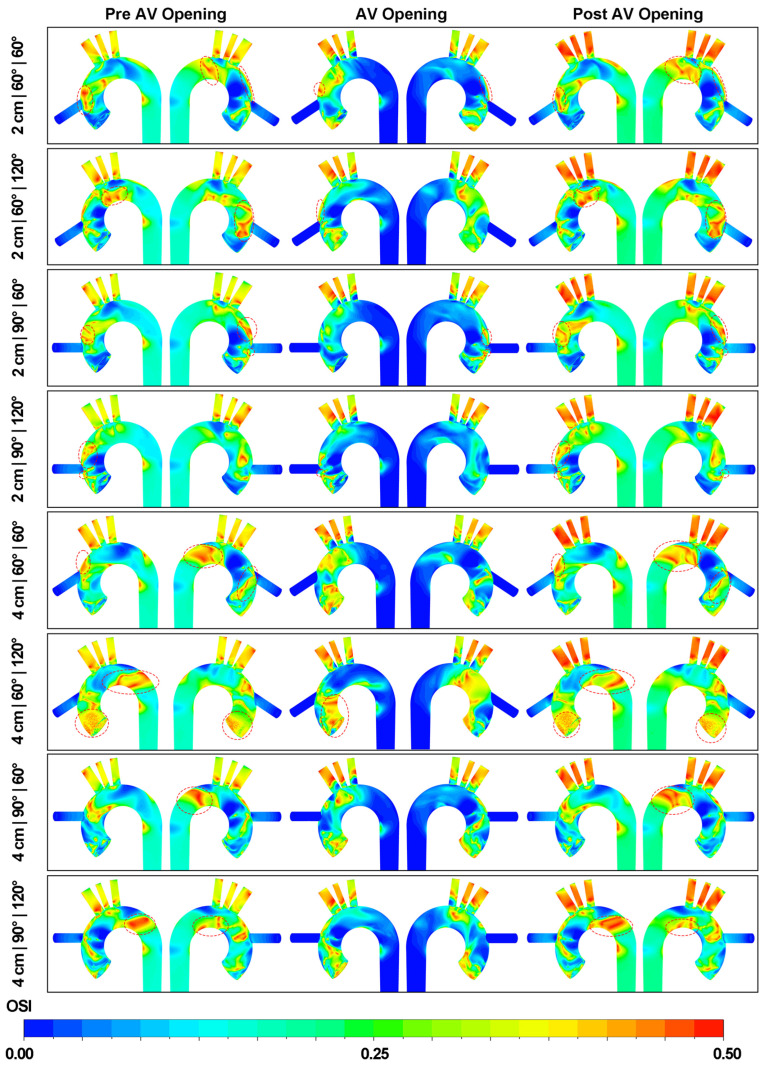
OSI distributions on the aortic wall at three different stages. The area with a dotted curve box on the aortic arch displays the high OSI region (OSI > 0.4).

Figure 12 illustrates the OSI distribution on the aortic wall. The OSI contours for each phase of the cardiac cycle are distinctly displayed, allowing for a comprehensive understanding of OSI variations throughout the cardiac cycle. When examined closely, the OSI values for the three epiaortic arteries do not remain consistently high throughout the entire cardiac cycle. In particular, the magnitude of OSI on the arteries is relatively lower during the acceleration of the graft velocity compared to its deceleration. The region around the graft flow impingement site maintains low OSI values, which suggests consistent flow direction at this place. It is also noted that localized regions of high OSI values emerge at the anterior or posterior sides of the distal aortic arch, which is contingent on varying azimuthal angles.

**Figure 13:**
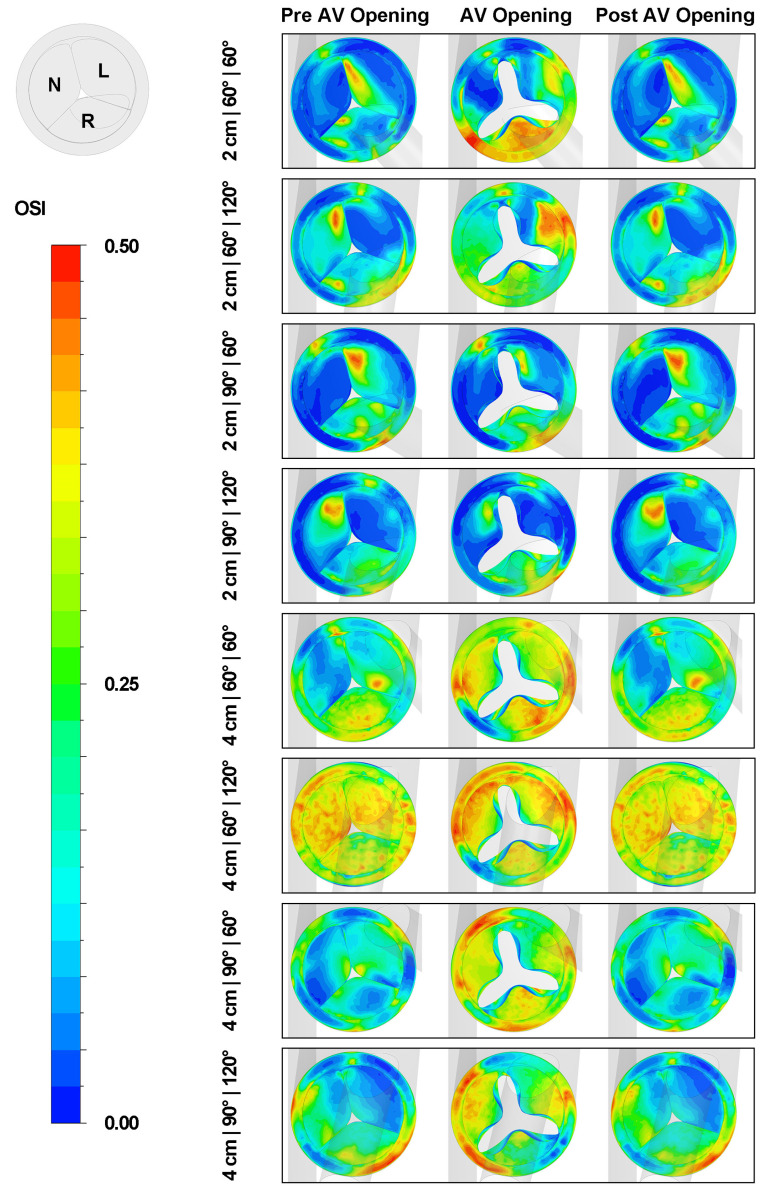
OSI distributions on aortic valve at three different stages. N: the non-coronary cusp. L: the left coronary cusp. R: the right coronary cusp.

Figure 13 presents the OSI distribution on the aortic valve. When the graft height is set at 2 cm, a region exhibiting high OSI values (OSI > 0.4) can be found on either the non-coronary cusp or the left coronary cusp. As the LVAD outflow height increases, a corresponding increase in the OSI value on the aortic valve is observed. This trend is particularly noticeable when comparing the 2 cm|60°|120° and 4 cm|60°|120° cases. Upon the opening of the aortic valve, there is a substantial increase observed in the OSI distribution on the right coronary cusp. This phenomenon can be attributed to the recirculation flow that occurs above the cusp. Concurrently, the OSI magnitudes on the remaining two cusps also elevate to varying degrees. When the aortic valve returns to the closed state, the OSI distribution on the aortic valve essentially reverts to its initial pattern, as seen prior to the opening of the aortic valve.

## 4. Discussion

This study employs multiple CFD simulations to comprehensively investigate the impact of different LVAD outflow cannula locations on aortic hemodynamics, which informs cardiac surgeons about technical aspects that need to be considered during LVAD implantation. It is the first computational study to report on the fluid dynamics on the aortic valve under LVAD support with intermittent valve opening. Our findings provide valuable insights into the mechanisms of aortic insufficiency as a valvular complication in LVAD patients. We identified various geometric factors associated with retrograde flow to the aortic valve and flow rotation in the aortic arch. Specifically, the study reveals that height and inclination angles influence the recirculation region at the aortic root, while the azimuthal angle determines blood flow rotation in the aortic arch. Further details are presented in the following subsections.

### 4.1. Aortic Valve Morphology

The incorporation of aortic valve morphology into hemodynamic analysis provides valuable insights, particularly at the aortic root when the valve is opening. Blood flow passes through the aortic valve forms a vortex ring along the direction of flow, which aligns the recirculation of flow lines above the valve. This contributes to a reduction in TAWSS on the aortic valve, which reduces the retrograde flow, flow velocity and shear rate on the aortic valve. The resulting, more stable shear conditions promote a protective response by increasing anti-inflammatory and anti-oxidative protein synthesis in valvular endothelial cells, as reported in a previous study [49]. Research has demonstrated that exposing aortic valve leaflets to supra-physiological oscillatory shear stress magnitudes can lead to chronic valvular inflammation [50], aortic valve remodeling and calcific aortic valve disease [50,51]. With time, the vortex ring gradually dissipates, giving rise to smaller eddies that coil around the graft flow. This observation explains the presence of distinct areas with a high OSI magnitude around the entrance of the LVAD outflow cannula on the aortic arch. These regions with elevated OSI have been closely associated with the formation of plaques and vascular wall-thickening in the aorta [52,53,54].

### 4.2. Height of LVAD Outflow Graft

Previous research has investigated the influence of LVAD outflow cannula height, albeit with a fixed insertion angle and an assumption of laminar flow in the aortic arch [22]. In this study, the height of the LVAD outflow cannula refers to the perpendicular distance from the graft entrance to the aortic root. Positioning the graft higher (i.e., closer to the proximal aortic arch) causes notable alternations in blood flow dynamics. This modification redirects more blood from the graft towards the center of the aortic arch and effectively reduces retrograde flow to the aortic root. Consequently, the TAWSS magnitude on the aortic valve is reduced, which is critical in preventing the progression of aortic valve calcification and eventual aortic insufficiency [49,50].

However, solely increasing the height of the outflow graft may not always provide an optimal solution for graft implantation. As the height of the LVAD outflow graft increases, the resulting vortex ring induced by the graft jet flow also shifts upwards, moving the vortex ring away from the aortic valve and reducing the number of eddies observed at the aortic root. Thus, blood at the aortic root becomes more stagnant, which increases the propensity for thrombogenesis [55]. In situations where there is less retrograde blood flowing at the aortic root (especially in the 4 cm|60°|120° case), the aortic root and the surrounding aortic wall experience much lower shear stress and a higher OSI magnitude. Maintaining an extremely low TAWSS level that is continually less than 0.2 Pa is not advantageous, as it can increase the likelihood of monocyte adherence to vascular endothelial cells and accentuate the risk of thrombosis as well [56]. Additionally, the combination of low TAWSS and high OSI levels deactivates the atheroprotective endothelial phenotype, ultimately leading to the progression of atherosclerosis [57,58]. Moreover, the blood flowing in the proximal aortic arch exhibits a relatively high velocity, leading to a secondary impingement just prior to blood entry into the descending aorta. Therefore, a significant increase in OSI magnitude is observed at the distal aortic arch, potentially contributing to type-B aortic dissection over time [59].

### 4.3. Inclination Angle of LVAD Outflow Graft

Two distinct inclination angles associated with the LVAD outflow graft have been studied and analyzed in this study. These specific angles were deliberately chosen to be less than 90 degrees, as a larger angle could result in increased retrograde flow to the aortic root. Such an increase in retrograde flow may elevate the transvalvular pressure to pathological levels, potentially leading to detrimental effects [21,23]. An intriguing finding is that alterations in the inclination angle exhibited similar effects to those observed with graft height adjustments in terms of modifying the separation of blood flow after impingement on the aortic wall. A smaller inclination angle directed a greater proportion of blood flow into the top lumen of the aortic arch. However, as the inclination angle gradually increased and approached the 90-degree mark, a stronger and more direct impingement on the aortic wall was observed, resulting in a larger reverse-flow region downstream from the anastomosis. This leads to an elevation in the magnitude of TAWSS on the aortic valve and aortic wall, which can potentially increase the expression of inflammation markers in valvular endothelial cells [60,61]. As previously mentioned, the inflammatory response triggered by the altered hemodynamics on the aortic valve plays a crucial role in the remodeling of the aortic valve. A proteomic study also identified a strong negative correlation between TAWSS and the level of proteins related to cellular structure and extracellular junctions [62]. This remodeling process leads to commissural fusion of the leaflets and a subsequent degenerative change in the aortic valve, which further contributes to the progression of aortic insufficiency and its associated clinical consequences [50].

Interestingly, the inclination angle can interact synergistically with the graft height, either amplifying or alleviating its influence. However, no significant changes were observed in the OSI magnitude with different inclination angles. This finding suggests that the inclination angle may have a lesser impact on the turbulence of blood flow within the aorta. Therefore, in situations where the LVAD outflow graft is positioned too high to effectively wash out the stagnant flow at the aortic root, adopting a larger inclination angle can serve as a potential mitigation strategy.

### 4.4. Azimuthal Angle of LVAD Outflow Graft

In contrast to the other two geometric factors, the azimuthal angle did not significantly impact the volume of retrograde flow directed towards the aortic root. However, we found that the azimuthal angle was related to the swirling direction of blood flow in the aorta. A smaller azimuthal angle guides the blood flow in a right-handed rotation along the aortic arch, while angles exceeding 90 degrees result in a predominantly left-handed flow direction. Exceptions are observed in cases with a lower height and larger inclination angles (2 cm|90°|60° and 2 cm|90°|120° cases), where the torsion of the aortic arch reduces the hemodynamic influence of the proximal aortic anastomosis, leading to a right-handed blood flow similar to native physiological conditions.

Additionally, the azimuthal angle influences the spatial distribution around the entrance of the LVAD outflow cannula. With an azimuthal angle of 60 degrees, the anterior part of the ascending aorta has ample space, while the posterior part becomes more spacious, with an azimuthal angle of 120 degrees. Consequently, the strong vertical vortex in the ascending aorta rotates in opposite directions due to this asymmetrical space distribution. During the valve opening stage, the eddies following the breaking of the vortex ring above the aortic valve also rotate in different directions, influenced by varying azimuthal angles. This leads to different regions on the aortic wall with a higher risk of plaque formation, as indicated by the high-OSI regions [52,53,54]. To maintain a hemodynamic environment closer to normal, a smaller azimuthal angle is recommended.

### 4.5. Limitations

We acknowledge several limitations of this study. First, the study focuses on the overall aortic structure, rather than specifically incorporating the geometry of the aortic sinuses, when constructing the aorta model. Additionally, the movement of the aortic valve during the cardiac cycle is not modeled as a continuous process but rather approximated through alternations in geometry. In this study, the wall of the aorta geometry is assumed to be rigid with no deformation. For future investigations, a more comprehensive approach can involve fluid–structure interactions (FSIs) to consider the elasticity of the aortic wall and acquire more accurate results. Another limitation lies in the small sample size of the LVAD outflow cannula geometry factors. The study examined two values for each geometric factor, which result in, at most, eight different LVAD outflow cannula locations. To obtain a more precise and comprehensive conclusion, a broader range of simulation cases encompassing various LVAD outflow graft insertion positions would be beneficial. Furthermore, this study does not consider the influence of blood rheology across the pathophysiological envelope. Patients with heart failure may have accompanying co-morbidities that affect blood viscosity and this may potentially affect the flow dynamics by reducing the Reynolds number. Furthermore, it is important to note that the findings of this study are based on simulations and require validation from advanced clinical imaging techniques such as echocardiographic vector flow mapping [63].

## 5. Conclusions

In this CFD study, we found that height and inclination angle play similar roles in controlling the amount of retrograde flow directed towards the aortic root following jet flow impingement. Conversely, the azimuthal angle predominantly determines the rotational direction of blood flow in the aortic arch. Therefore, during the LVAD implantation procedure, the precise positioning of the LVAD outflow graft emerges as a critical determinant to optimize patient outcomes. Moreover, the incorporation of valve morphology enables the study of hemodynamic parameters on the aortic valve under LVAD support, providing valuable insights into the development of aortic insufficiency after LVAD implantation. However, further evaluation of the aortic valve following long-term LVAD support is necessary to validate and reinforce the findings acquired from this study.

## Figures and Tables

**Table 1:** Eight LVAD outflow graft configurations.

Case	Height (cm)	Inclination Angle (Degree)	Azimuthal Angle (Degree)
2 cm|60°|60°	2	60	60
2 cm|60°|120°	2	60	120
2 cm|90°|60°	2	90	60
2 cm|90°|120°	2	90	120
4 cm|60°|60°	4	60	60
4 cm|60°|120°	4	60	120
4 cm|90°|60°	4	90	60
4 cm|60°|120°	4	90	120

**Table 2:** Three-element Windkessel model parameters for each pressure outlet.

	Windkessel Resistance $R_{C}$ $Pa\cdot s\cdot m^{-3}$	Windkessel Compliance C $m^{3}\cdot {Pa}^{-1}$	Windkessel Resistance $R_{P}$ $Pa\cdot s\cdot m^{-3}$
BCA	$5.1918\cdot 10^{7}$	$8.6974\cdot 10^{-10}$	$10.608\cdot 10^{8}$
LCCA	$19.1515\cdot 10^{7}$	$1.7670\cdot 10^{-10}$	$52.2129\cdot 10^{8}$
LSA	$9.8820\cdot 10^{7}$	$7.0871\cdot 10^{-10}$	$13.0183\cdot 10^{8}$
Descending aorta	$1.1752\cdot 10^{7}$	$1.0163\cdot 10^{8}$	$1.1167\cdot 10^{8}$

## Data Availability

No new data were created or analyzed in this study. Data sharing is not applicable.

## Supplementary Materials

The following supporting information can be downloaded at: https://www.mdpi.com/article/10.3390/biomimetics8060465/s1, Figures S1–S5: Secondary velocity contour in cross-sectional view.
