# Supplementary material for: The Impact of Left Ventricular Assist Device Outflow Graft Positioning on Aortic Hemodynamics: Improving Flow Dynamics to Mitigate Aortic Insufficiency
Source: Biomimetics (Basel). 2023 Oct 1;8(6):465. doi: 10.3390/biomimetics8060465 (PMC10604423; doi:10.3390/biomimetics8060465)
Supplement: Supplementary file 1 [file biomimetics-08-00465-s001.zip › biomimetics-2556472-supplementary.pdf]

Supporting Information

Supporting Figures:

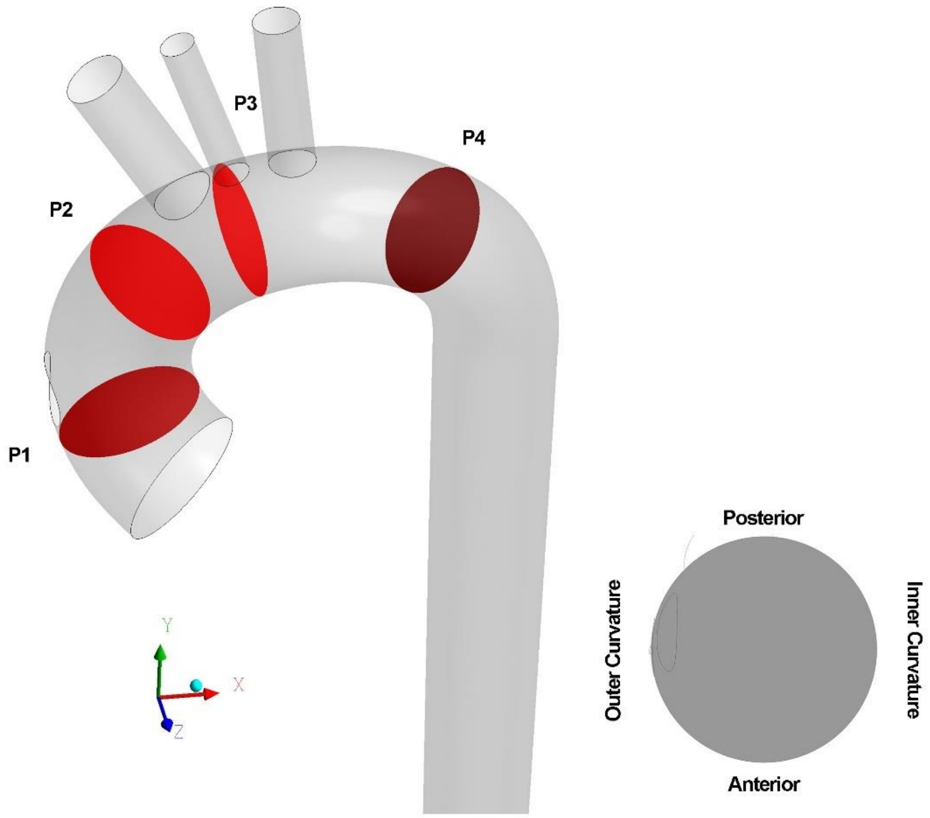

Figure S1: Illustration of the four cross section planes in the fluid domain

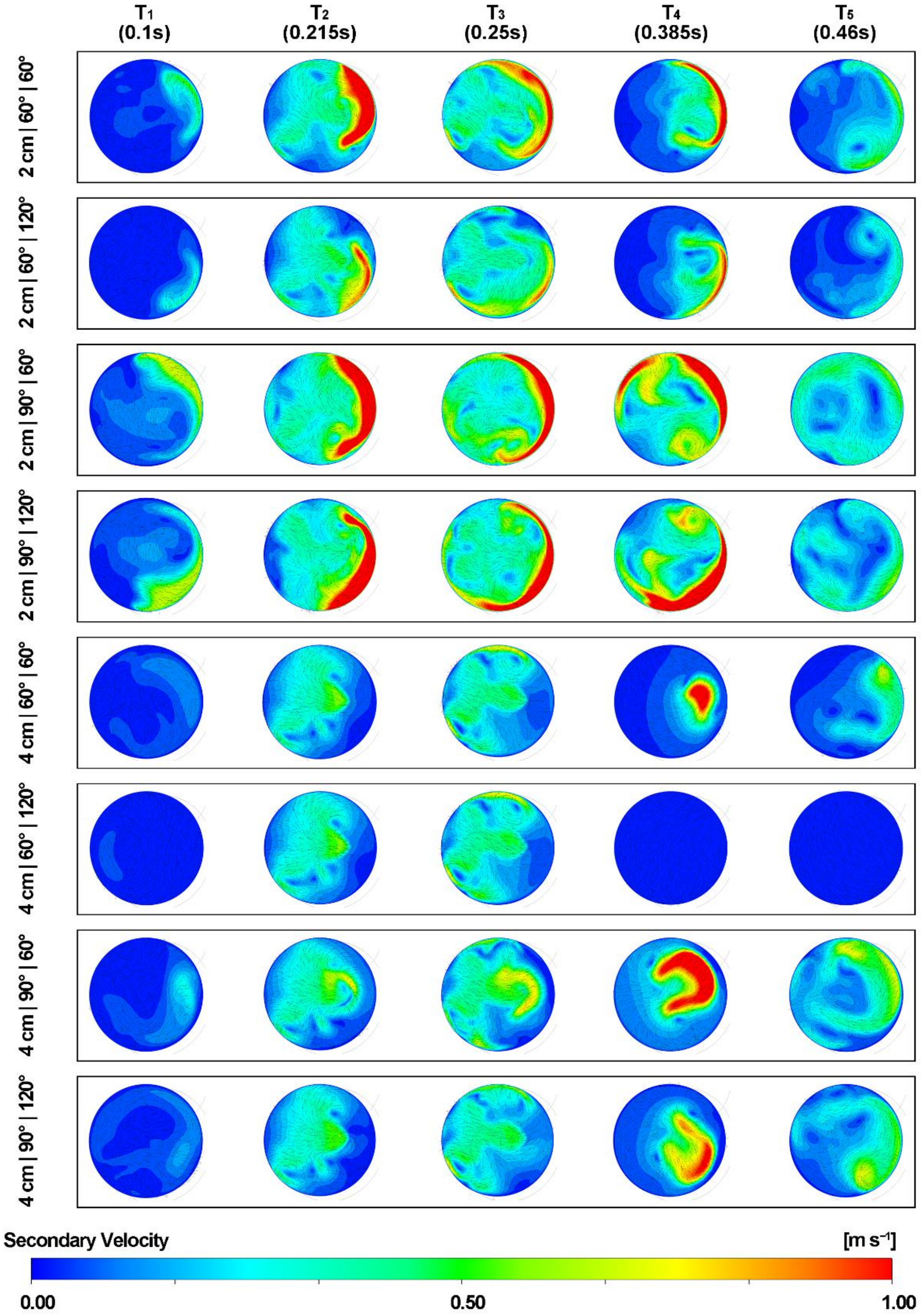

Figure S2: Secondary velocity contour at P1

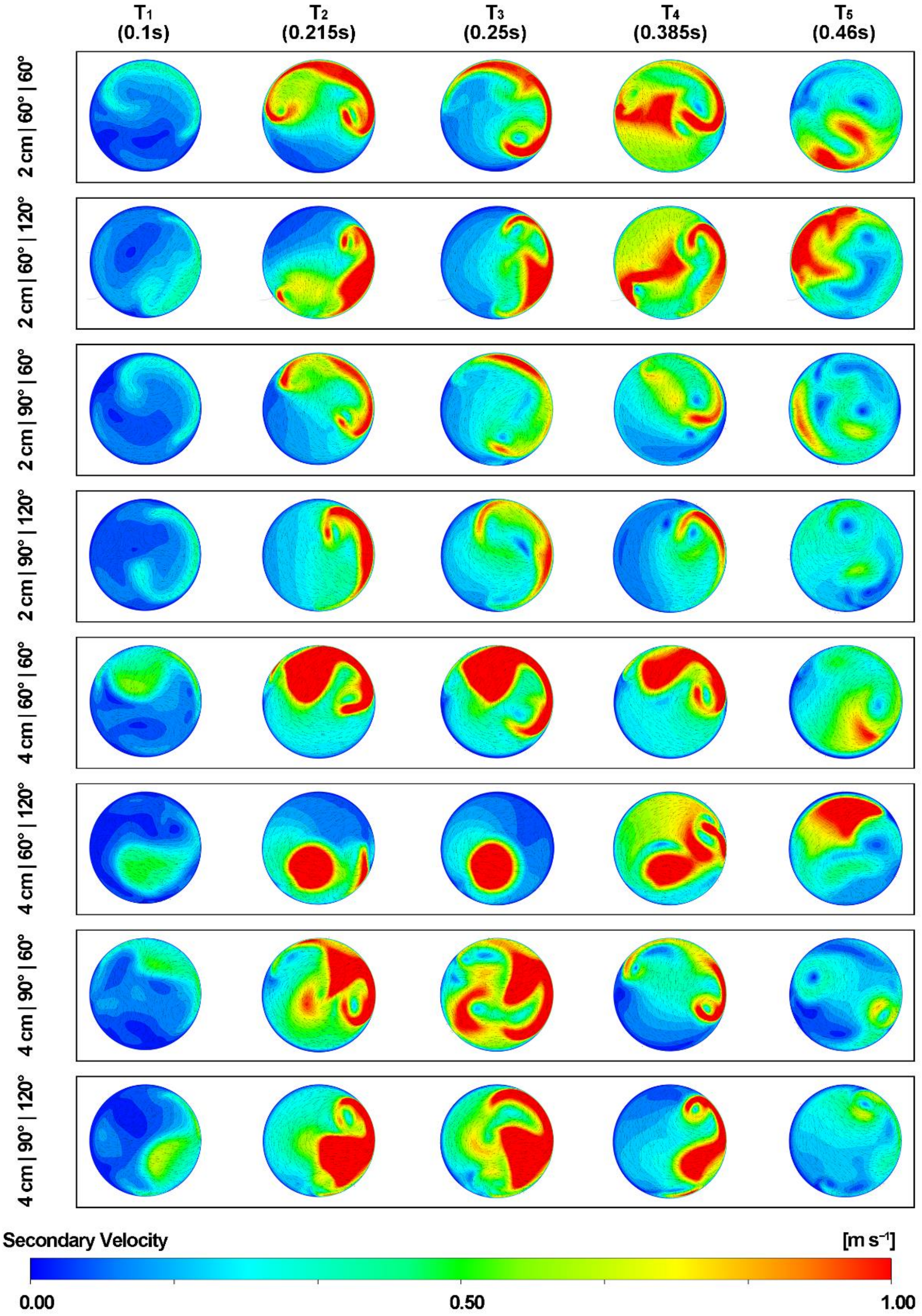

Figure S3: Secondary velocity contour at P2

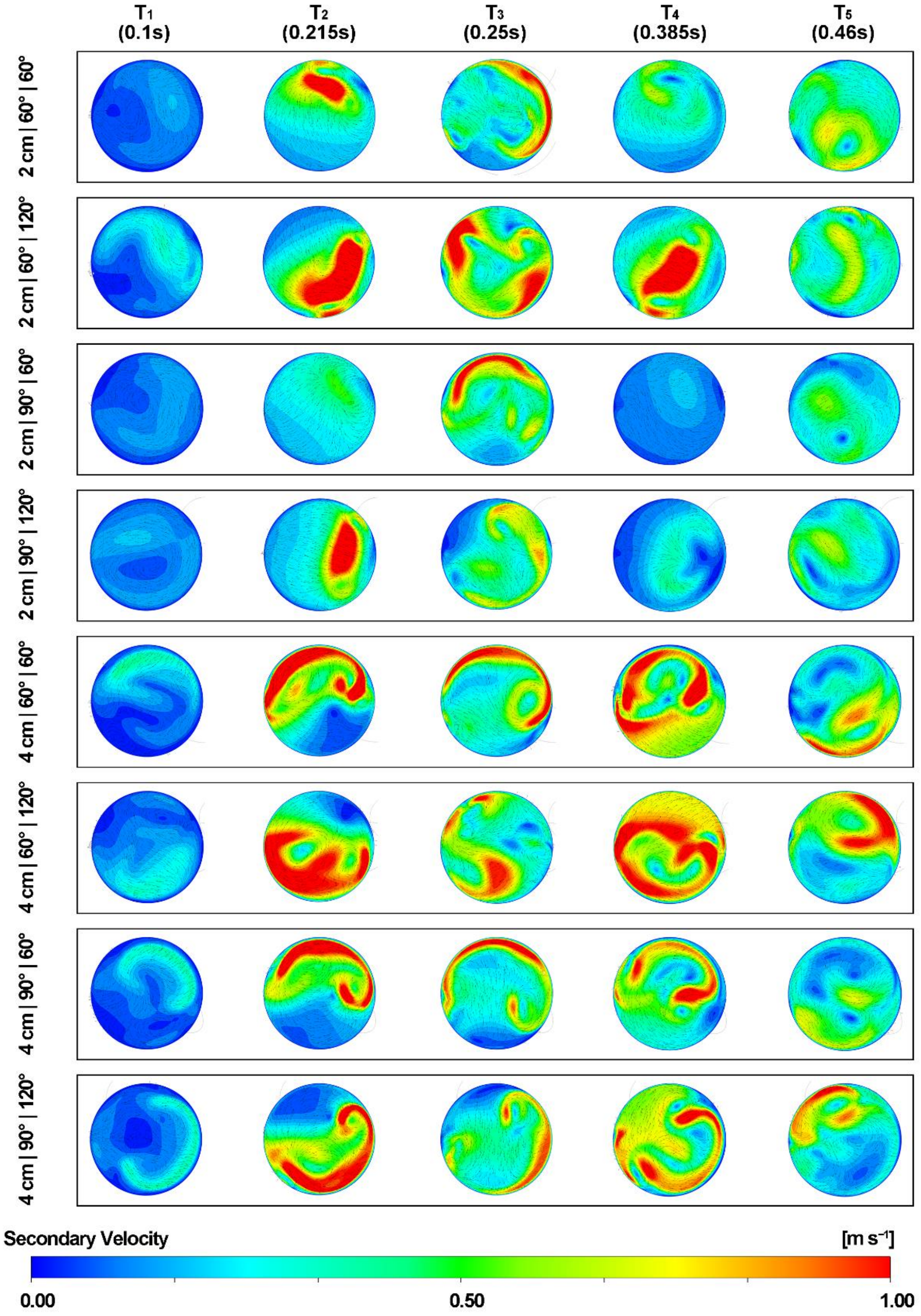

Figure S4: Secondary velocity contour at P3

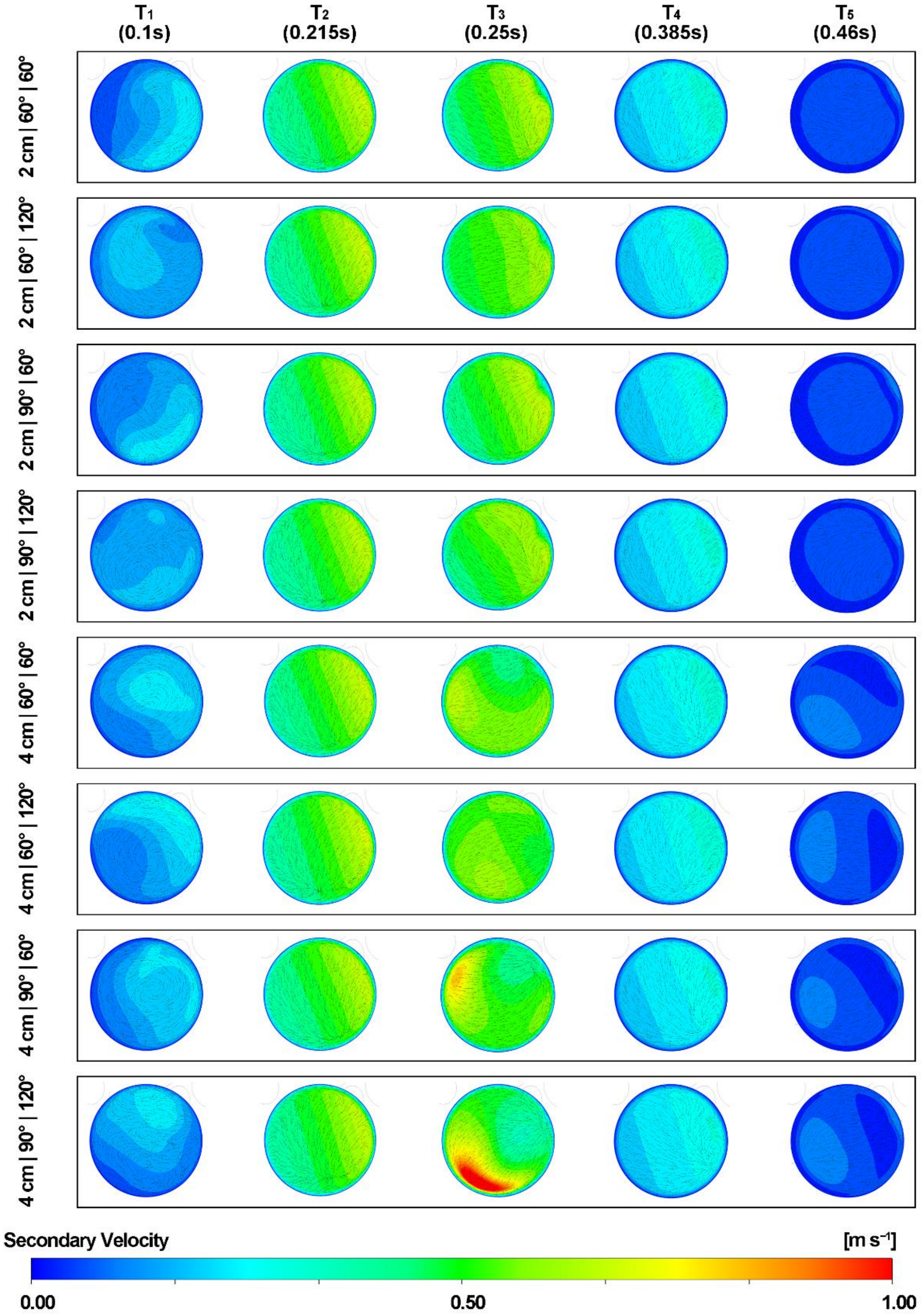

Figure S5: Secondary velocity contour at P4
